# The effect of sarcopenia and sarcopenic obesity on survival in gastric cancer

**DOI:** 10.1186/s12885-023-11423-y

**Published:** 2023-09-28

**Authors:** Muzaffer Ece Hakan Şahin, Feray Akbaş, Aytul Hande Yardimci, Eren Şahin

**Affiliations:** 1 Clinic of Internal Medicine, Ministry of Health Serik State Hospital, 07500 Antalya, Turkey; 2 Department of Internal Medicine, University of Health Sciences, İstanbul Training and Research Hospital, 34098 Fatih Istanbul, Turkey; 3 Aytul Hande Yardimci, Department of Radiology, University of Health Sciences, Başakşehir Cam and Sakura City Hospital, 34480 Başakşehir Istanbul, Turkey; 4 Faculty of Medicine, Department of Medical Oncology, Akdeniz University, 07070 Konyaaltı Antalya, Turkey

**Keywords:** Body mass index, Computerized tomography, Gastric cancer, Obesity, Sarcopenia, Sarcopenic obesity,Skeletal muscle index

## Abstract

**Background:**

Sarcopenic obesity arises from increased muscle catabolism triggered by inflammation and inactivity. Its significance lies in its role in contributing to morbidity and mortality in gastric cancer. This study aims to explore the potential correlation between sarcopenia, sarcopenic obesity, and gastric cancer, as well as their effect on survival.

**Materials and methods:**

This retrospective study included 162 patients aged ≥ 18 years who were diagnosed with stomach cancer. Patient age, gender, diagnostic laboratory results, and cancer characteristics were documented. Sarcopenia was assessed using the skeletal muscle index (SMI) (cm2/m2), calculated by measuring muscle mass area from a cross-sectional image at the L3 vertebra level of computed tomography (CT).

**Results:**

Among the 162 patients, 52.5% exhibited sarcopenia (with cut-off limits of 52.4 cm2/m2 for males and 38.5 cm2/m2 for females), and 4.9% showed sarcopenic obesity. Average skeletal muscle area (SMA) was 146.8 cm2; SMI was 50.6 cm2/m2 in men and 96.9 cm2 and 40.6 cm2/m2 in women, respectively. Sarcopenia significantly reduced mean survival (*p* = 0.033). There was no association between sarcopenic obesity and mortality (*p* > 0.05), but mortality was higher in sarcopenic obesity patients (*p* = 0.041). Patient weight acted as a protective factor against mortality, supporting the obesity paradox. Tumor characteristics, metabolic parameters, and concurrent comorbidities did not significantly impact sarcopenia or mortality.

**Conclusion:**

Sarcopenia is more prevalent in the elderly population and is linked to increased mortality in gastric cancer patients. Paradoxically, higher body mass index (BMI) was associated with improved survival. Computed tomography offers a practical and reliable method for measuring muscle mass and distinguishing these distinctions.

**Trial registration:**

This study was approved by Istanbul Training and Research Hospital Clinical Research Ethics Committee of the University of Health Sciences (29.05.2020/2383).

## Background

Sarcopenia is described as progressive and generalized loss of muscle mass, strength or function. Primary sarcopenia is a consequence of the aging process preceding frailty, while secondary sarcopenia is linked to factors like inactivity, poor nutrition, comorbidities such as organ failure, chronic inflammation, or cancer [1–3].

Various diagnostic methods are available for sarcopenia, including nuclear magnetic resonance imaging (MRI), computed tomography (CT), dual-energy X-ray absorptiometry (DEXA), bioelectrical impedance analysis for muscle mass, and tests for muscle function like gait speed, hand-grip strength, and low extremity strength [4–7]. Thus, it can easily be screened and diagnosed with a variety of tools and methods, most of which are simple and readily available.

Sarcopenic obesity is a recently coined term that describes the combination of sarcopenia and obesity, characterized by elevated body mass index (BMI), waist circumference (WC), total fat mass, body fat percentage, or visceral fat area [4]. It arises due to increased muscle catabolism triggered by inflammation and/or inactivity in patients with obesity [8]. Both sarcopenia and sarcopenic obesity are associated with higher overall mortality in cancer patients [9], rendering them valuable prognostic indicators in malignancy cases.

Gastric cancer, ranking fifth in terms of prevalence and third in cancer-related deaths [10], has been implicated in a reciprocal relationship with sarcopenia, possibly contributing to its development and progression. While the precise mechanisms remain unclear, muscle wasting in sarcopenia is believed to interact with systemic inflammation, altered immune function, and nutritional deficits, potentially facilitating cancer growth and metastasis [11]. Conversely, gastric cancer-related inflammation and metabolic changes may exacerbate sarcopenia [1, 12]. Regardless of the definition and measurement method employed, sarcopenia is linked to poor prognosis in gastric cancer [13–15].

In this context, this study aims to explore the relationship between gastric cancer and sarcopenia/sarcopenic obesity, investigating their potential as predictive tools for mortality in this patient group, as well as the additional benefits of CT for detecting these conditions.

## Materials and methods

### Patient population

The study included adult patients diagnosed with gastric cancer at our hospital between 2015 and 2019, adhering to inclusion and exclusion criteria.

Inclusion criteria comprised age ≥ 18, any gender, presence of a medical file in our oncology clinic, availability of height-weight data, and an abdominal CT scan covering the entire abdomen and the third lumbar spine level, conducted prior to treatment.

Exclusion criteria encompassed patients lacking BMI data, those with only post-operative CT scans, and patients whose CT scans did not include the third lumbar spine level.

A digital database was established for laboratory values, tumor histopathological features, treatment protocols, complications, and dates of diagnosis and death. A computer program (PROBEL) facilitated data processing. Out of 1136 patients diagnosed with malignant neoplasm of the stomach (C16) in our system, 162 met the study's criteria. Non-sarcopenic patients were selected as the control group.

Patients were categorized based on concomitant comorbidities and treatment-related complications. Lauren's classification was used for adenocarcinomas [16], and the WHO classification was employed for cancer types [17]. Tumor locations were classified as esophagogastric junction, cardia, corpus, antrum, and pylorus. Treatment modalities included chemotherapy, radiotherapy, and surgery. Subtotal and partial gastrectomy were grouped together, along with the wedge resection category comprising patients undergoing mass excision.

### Imaging analysis

**Fig. 1:**
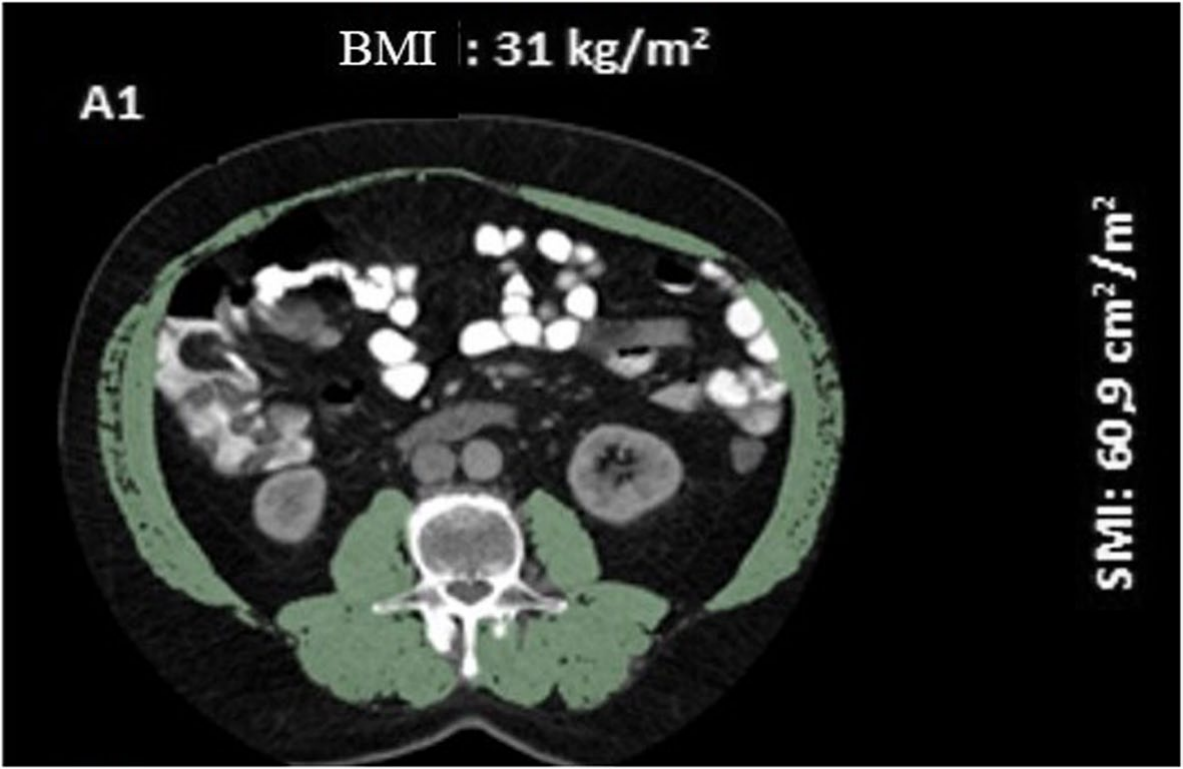
Example of skeletal muscle index measurements on CT sections through the L3 vertebra level. A1: BMI 31 kg/m2 and SMI 60,9 cm2 /m2

CT examinations were performed using a 64-detector scanner (Aquilion, Canon Medical Systems, Japan) and a 128-detector scanner (Philips, Marifet, The Netherlands), with analysis carried out using the 3D Slicer (Version 4.10.2) open-source software program. Segmentation analysis was conducted on a single slice where the transverse processes of the L3 vertebra corpus were visiblein the axial plane [18, 19]. The muscles were specified as psoas, erector spinae, quadratus lumborum, transversus abdominis, external and internal oblique, and rectus abdominis. Measurements were made by tissue-specific threshold technique (between -30 and 130), and a masking method was used on selected images accordingly. Muscle tissues were segmented on CT images, and the results obtained by quantification were extracted for each patient. The cross-sectional muscle area was normalized to the patient's height to calculate the skeletal muscle index (SMI) (Fig. 1).

The presence of sarcopenia was determined by dividing SMA value (calculated on CT) by the square of the patient's height (m2) and was expressed as SMI, with cutoff values set at 52.4 cm2/m2 for men and 38.5 cm2/m2 for women based on previous large-scale studies [20].

Patients were categorized by BMI (weight/(height)2) as underweight (BMI < 18.5 kg/m2), normal weight (18.5–24.9 kg/m2), overweight (25–29 kg/m2), and having obesity (≥ 30 kg/m2). Sarcopenic obesity was defined as the coexistence of sarcopenia and obesity in patients with a BMI ≥ 25 kg/m2 [10].

### Statistical analysis

SPSS 22.0 program was used in the analysis. We used mean, standard deviation, median lowest, highest, frequency, and ratio values in the descriptive statistics of the data. Independent t-test or Mann–Whitney U tests were used in the analysis of quantitative independent data, and the chi-square test was used for the analysis of qualitative independent data. Multivariate Cox regression (hazard regression) was used to calculate the effect of skeletal muscle index on survival. Univariate regression analysis was used to calculate the survival effect of all variables. A *p*-value of < 0.05 was considered statistically significant for all analyses.

## Results

### Demographic and clinical descriptive features

The study included 162 patients; 66.7% (*n* = 108) were male. The mean age was 63.3 ± 10.3 years, and 52.5% (*n* = 85) were ≥ 65 years old. BMI distribution was as 2.5% (*n* = 4) low, 40.1% (*n* = 65) normal, 32% (*n* = 52) overweight, and 25.3% (*n* = 41) with obesity. When overweight and obesity (BMI ≥ 25 kg/m2) were evaluated together, obesity prevalence was 34.5% (*n* = 56) (Table 1).

**Table 1:** The demographic data of patients with clinical features

Variables		n	%
Gender	Male	108	(66,7)
	Female	54	(33,3)
Age	< 65	77	(47,5)
	≥ 65	85	(52,5)
BMI	Underweight	4	(2,5)
	Normal weight	65	(40,1)
	Overweight	52	(32,1)
	Obesity	41	(25,3)
Lauren Classification	Intestinal	46	(28,4)
	Diffuse	37	(22,8)
	Indeterminate	65	(40,1)
	Non-adenocarcinoma	14	(8,6)
WHO Classification	Papillary	3	(1,9)
	Tubular	27	(16,7)
	Mucinous	14	(8,6)
	Signet ring & poorly cohesive	34	(21,0)
	Mixed	84	(51,9)
Tumor Stage	Stage	18	(11,1)
	Stage 2	44	(27,2)
	Stage 3	50	(30,9)
	Stage 4	50	(30,9)
Tumor Location	Non-cardia	109	(67,3)
	Cardia	53	(32,7)
Comorbidity			
Diabetes Mellitus	-	129	(79,6)
	+	33	(20,4)
Cardiovascular disease	-	80	(49,4)
	+	82	(50,6)
Hyperlipidemia	-	110	(67,9)
	+	52	(32,1)
Cancer	-	150	(92,6)
	+	12	(7,4)
Other comorbidities	-	137	(84,6)
Complication	-	58	(35,8)
	+	104	(64,2)
Surgery	-	45	(27,8)
	+	117	(72,2)
Type of surgery	-	45	(27,8)
	Total	81	(50,0)
	Subtotal	27	(16,7)
	Wedge	9	(5,6)
Chemotherapy	-	22	(13,6)
	+	140	(86,4)
Radiotherapy	-	100	(61,7)
	+	62	(38,3)
Total		162	(100)

Cancer types were intestinal (*n* = 46, 28.5%), diffuse (*n* = 37, 22.8%), indeterminate (*n* = 65, 40.1%), and non-adenocarcinoma (*n* = 14, 8.6%); tumors types were papillary (*n* = 3, 1.9%), tubular (*n* = 27, 16,7%), mucinous (*n* = 14, 8.6%), signet ring and weak cohesive (*n* = 14, 21%) and the mixed (*n* = 84, 51,9%). The tumors were stage 1 in 11.1% (*n* = 18), stage 2 in 27.2% (*n* = 44), stage 3 in 30.9% (*n* = 50), and stage 4 in 30.9% (*n* = 50) of the patients.

### The relationship of patient characteristics with sarcopenia

The mean SMA of < 65 years was 146 cm2 and SMI 50.4 cm2/m2, and the SMA of ≥ 65 years was 125.6 cm2 and SMI 46.6 cm2/m2. SMA and SMI values were higher for those < 65 ages (*p* = 0.001 and *p* = 0.006, respectively) and in men when compared to women (*p* < 0.001). The mean SMA was 146.8 cm2 SMI 50.6 cm2/m2for men, and 96.9 cm2 SMI 40.6 cm2/m2 for women. Sarcopenia was found in 52,5% (*n* = 85) of the patients; 61,1% (*n* = 66) were men. Sarcopenia was significantly higher (*p* = 0.003) > 65 ages and in men (*p* = 0.002) (Table 2).

**Table 2:** The Demographic data of the patients according to sarcopenia

Variables	Sarcopenia + *N* = 85	Sarcopenia - *N* = 77	*P* value
Age, < 65, n (%)	31 (40,3)	46 (59,7)	0,003
> 65, n (%)	54 (63,5)	31 (36,5)	
Male, n (%)	66 (61,1)	42 (38,9)	0,002
Female, n (%)	19 (35,2)	35 (64,8)	
BMI			0,001
Underweighted, n (%)	3 (75)	1 (25)	
Normal weight, n (%)	49 (75,4)	16 (24,6)	
Overweight, n (%)	25 (48,1)	27 (51,9)	
Obesity, n (%)	8 (19,5)	33 (80,5)	
DM			0,789
DM +	18 (54,2)	15 (45,5)	
DM -	67 (51,9)	62 (48,1)	
Hyperlipidemia			0,268
HL +	24 (46,2)	28 (53,8)	
HL -	61 (55,5)	49 (44,5)	
Cardiovascular disease (CVD)			0,349
CVD +	46 (56,1)	36 (43,9)	
CVD -	39 (48,8)	41 (51,3)	
Others (HBV + , COPD, CKD)			0,701
+	14 (56,0)	11 (44,0)	
-	71 (51,8)	66 (48,2)	
Total, n (%)	85 (52,5)	77 (47,5)	

*DM* Diabetes Mellitus, *CVD* Cardiovascular disease, *HBV* Hepatitis B virus, *COPD* Chronic obstructive pulmonary disease, *CKD* Chronic kidney disease

The mean age was 61 ± 11.2 years in non-sarcopenic and 65.4 ± 9 years, in sarcopenic patients. Increase in sarcopenia with age was statistically significant (*p* = 0.003). The mean BMI was 28.8 kg/m2 in patients without sarcopenia and 24.2 kg/m2 in patients with sarcopenia. There was a negative correlation between BMI and sarcopenia (*p* < 0.001).

Sarcopenia was found in 19.5% and 48.1% of the patients in the obesity and overweight groups, respectively and it was significantly higher in non-obesity patients (75.4%) (*p* < 0.001).No correlation was found between the existence of sarcopenia and laboratory parameters or co-morbid diseases in gastric cancer patients.

Sarcopenic patients were more likely to have cardia-located malignancies(*p* = 0.004). The complication rate after treatment was 64,2% (*n* = 104). There was no statistical significance for complication occurrence and undergoing surgery, chemotherapy, and radiotherapy in sarcopenic and non-sarcopenic patients.

### Patients and the treatment modalities (chemotherapy, partial gastrectomy and total gastrectomy)

In this cohort, 86.4% (*n* = 140) of the patients had chemotherapy and 13.6% (*n* = 22) did not. Sarcopenia was found in 52.9% (*n* = 74) of the patients who had chemotherapy and 50% (*n* = 11) of the patients who did not and the difference was not statistically significant (*p* = 0,803) (Table 3).

**Table 3:** The Relationship of treatment modality & complications and mortality & sarcopenia

		**Mortality**				***p***		**Sarcopenia**				***p***
		**Alive**		**Exitus**				**(-)**		**( +)**		
		**n**	**%**	**n**	**%**			**n**	**%**	**n**	**%**	
**Complications** **after treatment**	(-)	45	(77,6)	13	(22,4)	< 0,001	(-)	32	(55,2)	26	(44,8)	0,146
	( +)	35	(33,7)	69	(66,3)		( +)	45	(43,3)	59	(56,7)	
**Operation**	(-)	8	(17,8)	37	(82,2)	< 0,001	(-)	20	(44,4)	25	(55,6)	0,626
	( +)	72	(61,5)	45	(38,5)		( +)	57	(48,7)	60	(51,3)	
**Operation type**	(-)	8	(17,8)	37	(82,2)	< 0,001	(-)	20	(44,4)	25	(55,6)	0,956
	Total	45	(55,6)	36	(44,4)		Total	40	(49,4)	41	(50,6)	
	Subtotal	19	(70,4)	8	(29,6)		Subtotal	13	(48,1)	14	(51,9)	
	Wedge	8	(88,9)	1	(11,1)		Wedge	4	(44,4)	5	(55,6)	
**Chemotherapy**	(-)	10	(45,5)	12	(54,5)	0,692	(-)	11	(50,0)	11	(50,0)	0,803
	( +)	70	(50,0)	70	(50,0)		( +)	66	(47,1)	74	(52,9)	
**Radiotherapy**	(-)	50	(50,0)	50	(50,0)	0,842	(-)	50	(50,0)	50	(50,0)	0,424
	( +)	30	(48,4)	32	(51,6)		( +)	27	(43,5)	35	(56,5)	

51.3% (*n* = 60) of patients who had an operation had sarcopenia and 48.7% (*n* = 57) did not (totally 72.2%, *n* = 117). 50% (*n* = 81) of the patients had total gastrectomy.Operation increased survival rates but having an operation or type of operation did not have a significant relationship with sarcopenia (*p* = 0,956).

### The relationship of patient characteristics with mortality

The mortality was higher in the existence of sarcopenia (*p* = 0.012). Patients were alive in 40% of the sarcopenic and 59.7% of the non-sarcopenic patients. As BMI increased, mortality rates decreased (*p* = 0.019). Low albumin value was a statistically significant predictor of mortality in non-sarcopenic patients (*p* = 0.009) (Table 4).Complications increased mortality (*p* < 0.001); the surgical treatment group had lower mortality (*p* < 0.001) when compared to chemotherapy and radiotherapy (*p* = 0.692, *p* = 0.842) groups.

**Table 4:** The Relationship of sarcopenia and laboratory parameters with mortality

Parameters	Sarcopenia-						*p*	Sarcopenia +						*p*
	Alive			Ex				Alive			Ex			
	Median	25 Per	75 Per	Median	25 Per	75 Per		Median	25 Per	75 Per	Median	25 Per	75 Per	
Hemoglobin	11,8	9,8	13,3	11,5	9,3	13,5	0,571	11,7	9,7	13,2	11,7	9,6	12,9	0,774
Hematocrit	35,7	31,9	39,8	36,7	30,8	41,0	0,819	35,4	31,4	39,4	36,3	31,1	39,5	0,904
Neu/Lymphocyte	2,2	1,8	3,5	3,0	2,0	4,2	0,088	2,4	2,1	3,5	3,0	2,1	4,4	0,282
Albumin	3,9	3,4	4,2	3,4	2,9	4,0	**0,009**	3,7	3,4	4,0	3,6	3,2	4,1	0,478
Total Protein	6,8	6,2	7,1	6,4	5,7	7,2	0,284	6,6	6,1	7,2	6,7	6,1	7,2	0,606

The mortality was significantly higher in the esophagogastric location (*p* = 0.014). There was a positive correlation between mortality and Lauren classification (*p* = 0.004) but not with WHO classification (*p* = 0.362).The mortality increased significantly as tumor stage advanced, in both non-sarcopenic and sarcopenic groups (Table 5). According to the Lauren classification, the mortality rate in indeterminate type gastric cancer was higher in the sarcopenic patients.

**Table 5:** The relationship of sarcopenia and tumor features with mortality

Tumor Features		Sarcopenia-				*p*	Sarcopenia +				*p*
		Alive		Ex			Alive		Ex		
		n	%	n	%		n	%	n	%	
Lauren Classification	Intestinal	14	(63,6)	8	(36,4)	0,145	13	(54,2)	11	(45,8)	0,057
	Diffuse	11	(61,1)	7	(38,9)		6	(31,6)	13	(68,4)	
	Indeterminate	13	(46,4)	15	(53,6)		11	(29,7)	26	(70,3)	
	Non Adenocancer	8	(88,9)	1	(11,1)		4	(80,0)	1	(20,0)	
WHO Classification	Papillary	0	(,0)	1	(100,0)	0,670	1	(50,0)	1	(50,0)	0,235
	Tubular	10	(71,4)	4	(28,6)		7	(53,8)	6	(46,2)	
	Mucinous	4	(57,1)	3	(42,9)		4	(57,1)	3	(42,9)	
	Signet ring	10	(58,8)	7	(41,2)		3	(17,6)	14	(82,4)	
	Mixed	22	(57,9)	16	(42,1)		19	(41,3)	27	(58,7)	
Tumor Stage	Stage 1	11	(84,6)	2	(15,4)	0,001	4	(80,0)	1	(20,0)	< 0,001
	Stage 2	17	(81,0)	4	(19,0)		16	(69,6)	7	(30,4)	
	Stage 3	13	(56,5)	10	(43,5)		10	(37,0)	17	(63,0)	
	Stage 4	5	(25,0)	15	(75,0)		4	(13,3)	26	(86,7)	
Tumor Location	Non-cardia	32	(60,4)	21	(39,6)	0,865	24	(42,9)	32	(57,1)	0,455
	Cardia	14	(58,3)	10	(41,7)		10	(34,5)	19	(65,5)	

Mortality increased by 1.03 with age (95% C.I. 1.009–1.062) (*p* = 0,008). The mortality risk was 5,621 times higher in Stage III (95% C.I. 1,317–23,985) (*p* = 0,020) and 33,426 times higher in Stage IV (95% C.I. 7,495–149.073) (*p* < 0,001) compared to Stage I. Mortality was 0,9 times higher in the population with lower BMI (95% CI 0.858–0.984) (*p* = 0.016).

**Fig. 2:**
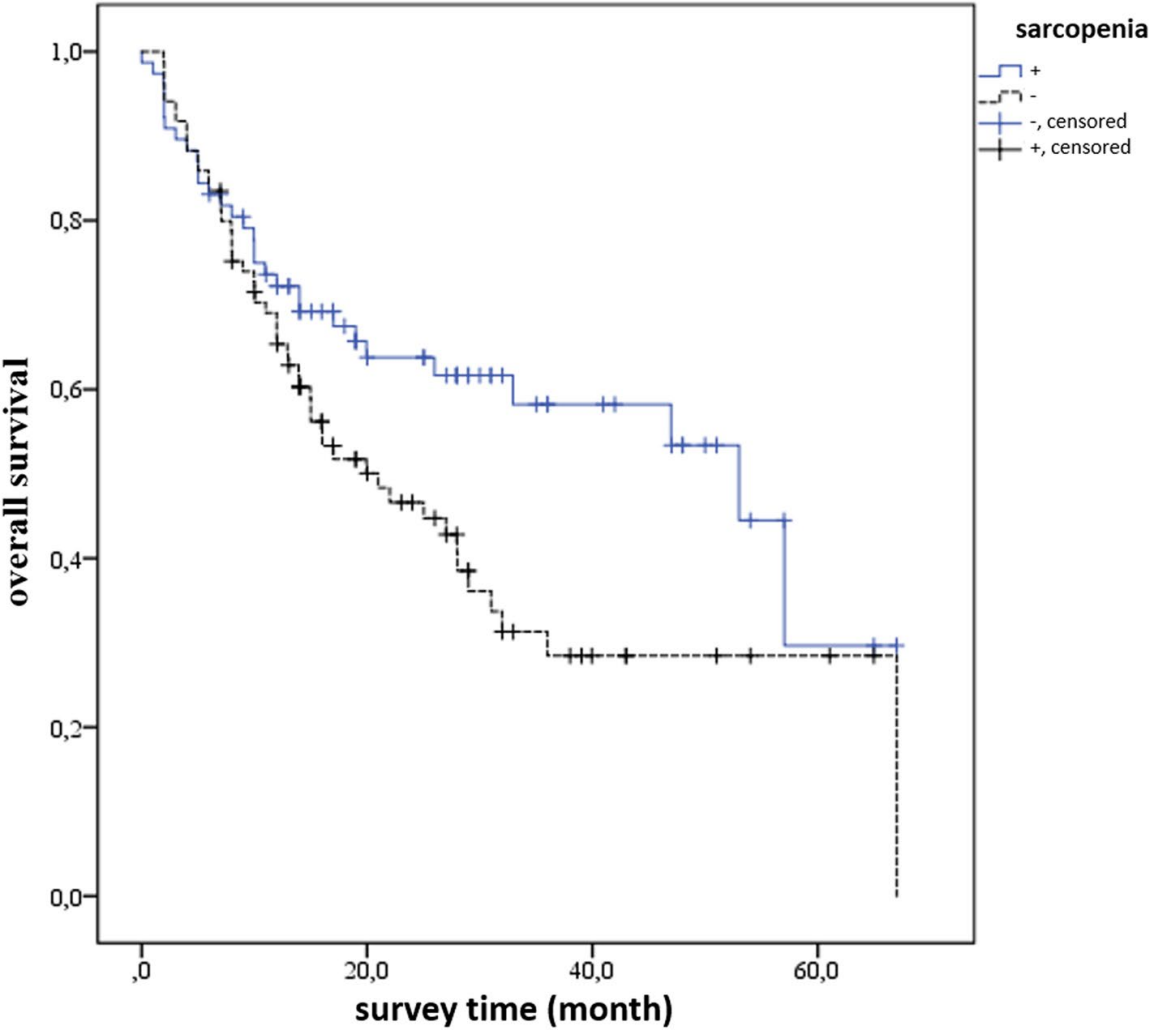
Survival curve according to sarcopenic status. The blue lines show the patient group without sarcopenia, and the black line indicates the sarcopenic patient group. Overall survival is shown as a percentage. Survival time is reduced in patients with sarcopenia

The mean survival time was 29.8 ± 3.1 and 39.9 ± 3.4 months in sarcopenic and non-sarcopenic patients, respectively. Sarcopenia significantly reduced the mean survival time (*p* = 0.033) (Fig. 2). There was no significant correlation between age, gender, obesity, WHO classification, and survival time with other locations except esophagogastric location (*p* > 0.005). The indeterminate subtype (mean 26.4 ± 3.5 months, C.I.:19,594–33.342) had a significantly lower survival time (*p* = 0.004). As the tumor stage advanced, the survival timesignificantly decreased (*p* < 0.001). The mean survival time in the esophagogastric location was significantly low (15.3 ± 3.6 months, *p* = 0.030).The correlation between sarcopenic obesity and mortality was insignificant (*p* > 0.05).

Also, cox regression analysis was made to evaluate the survival time. In univariate analysis; age (*p* = 0.011), BMI (*p* = 0.014), SMA (*p* = 0.021), SMI (*p* = 0.023), stage (*p* = 0.000), complication (*p* = 0.001), operation (*p* = 0.000) and RT (*p* = 0.049) had statistically significant effet on survival time. In multivariate analysis; age (*p* = 0.001), BMI (*p* = 0.001), SMA (*p* = 0.005), stage (*p* = 0.000) and operation (*p* = 0.000) had significant-independent effect on survival time (Table 6).

**Table 6:** Cox regression analysis of survival time

	Univariate Model					Multivariate Model				
	HR	% 95 CI			*p*	HR	% 95 CI			*p*
Age (years)	1.030	1.007	-	1.054	0.011	1.041	1.017	-	1.066	0.001
Gender	1.136	0.702	-	1.837	0.604					
BMI (kg/m2)	0.941	0.896	-	0.988	0.014	0.917	0.872	-	0.964	0.001
SMA	1.000	1.000	-	1.000	0.021	1.000	1.000	-	1.000	0.005
SMI	1.000	1.000	-	1.000	0.023					
Lauren	1.047	0.848	-	1.293	0.668					
WHO 2010	1.143	0.946	-	1.381	0.166					
Stage	3.112	2.279	-	4.251	0.000	1.952	1.351	-	2.821	0.000
Tm location	1.101	0.697	-	1.739	0.681					
DM	1.125	0.663	-	1.910	0.662					
CAD	1.486	0.953	-	2.318	0.081					
HL	0.939	0.587	-	1.503	0.794					
Cancer	0.429	0.156	-	1.183	0.102					
Hemoglobin	0.946	0.860	-	1.041	0.256					
Hct	0.979	0.944	-	1.016	0.263					
NLR	1.002	0.955	-	1.050	0.945					
Albumin	0.707	0.470	-	1.062	0.095					
Total protein	1.059	0.804	-	1.396	0.682					
Complication	2.783	1.532	-	5.055	0.001					
Operation	0.123	0.074	-	0.203	0.000	0.192	0.098	-	0.377	0.000
Operation type	0.222	0.148	-	0.333	0.000					
CTX	0.800	0.431	-	1.483	0.479					
RTX	0.626	0.393	-	0.998	0.049					

Cox Regresyon (Forward LR)

*BMI* Body Mass Index, *SMA* Skeletal Muscle Mass, *SMI* Skeletal Muscle Index, *DM* Diabetes Mellitus, *HT* Hypertension, *CAD* Coronary Artery Disease, *HL* Hyperlipidemia, *NLR* Neutrophil Lymphocyte Ratio, *CTX* Chemotherapy, *RTX* Radiotherapy)

### Association of sarcopenic obesity with research endpoints

In the examination performed by grouping the patients with overweight (BMI 25–29 kg/m2) and obesity (BMI ≥ 30 kg/m2) as "sarcopenic obesity", "sarcopenia + & obesity-", "sarcopenia- & obesity + " and "sarcopenia- & obesity-" the sarcopenic obesity group had a correlation with age and gender. Sarcopenic obesity was more prevalent in ≥ 65 years of age and common in men (*p* = 0.019 and *p* = 0.015, respectively).

There was apositive correlation between sarcopenic obesity patients and the presence of mortality and post-treatment complications. The post-treatment complication (*p* = 0.019) and mortality (*p* = 0.041) rates were higher in the sarcopenic obesity group. Those with obesity and sarcopenia had increased mortality compared to those who had obesitywithout sarcopenia (*p* = 0.041).

Non-cardia tumor localization was higher in the sarcopenic obesity group but not statistically significant. There was no statistically significant relationship between sarcopenic obesity and the tumor stage, tumor location or the presence of co-morbid disease.

## Discussion

The prevalence of sarcopenia in gastric cancer varies between 12.5% and 69.8% [10]. In our study, 52.5% of the patients were sarcopenic. The prevalence of sarcopenic obesity was 25.3% when BMI was ≥ 30 kg/m2 and 34.5% when BMI was ≥ 25 kg/m2. In a study examining the relationship between geriatric gastrointestinal cancers and sarcopenia, and sarcopenic obesity, the prevalence of sarcopenia was 30%, similar to our findings [21]. In a meta-analysis, sarcopenia had a prevalence of 10% in individuals aged ≥ 60 and increased to 20% when bioelectrical impedance analysis was used for diagnosis [22]. The differences in prevalence between studies may originate from using different methodologies such as DEXA, CT, and anthropometric and physical performance measures. There is no consensus yet on the exact numerical values for the assessment of sarcopenia.

Sarcopenia increases with age in the healthy population and is more common in patients over 65, as in the presented study [23]. Sarcopenic obesity was also higher in gastric cancer patients aged ≥ 65 years compared to sarcopenic patients without obesity or other non-sarcopenic patient groups, similar to previous studies [24]. Batsis et al. [25] reported the prevalence of sarcopenic obesity as 42.9% in men and 18.1% in women in a population ≥ 60 years old, supporting the presence of sarcopenia in gastric cancer patients increasing with age.

As in ours, sarcopenia is more common [18, 26, 27] and associated with fatigue in men [28]. Hypogonadism developing with cancer in men is assumed to have more negative effects than in women [20]. In advanced cancers, hypogonadism is associated with low muscle mass, fatigue, decreased physical activity, and survival [29, 30]. Testosterone replacement to prevent the loss of muscle mass due to cancer is still controversial [31]. Kim et al. [32] showed that obesity increased early in gastric cancer and well-to-moderately differentiated adenocarcinoma in men. Obesity was associated with dysplasia that develops independently of H. pylori infection in women. We found that 76% of the men with obesity were in the non-sarcopenic group. In the cohort, 51% of men and 68.5% of women had obesity. There was no correlation between tumor stage and obesity for genders.

In a study on solid tumors involving the respiratory system and gastrointestinal tract, 15% of 250 patients with obesity had sarcopenia, but 85% did not [18]. Sarcopenic obesity was more common in men, patients aged ≥ 65 years, and those with colorectal cancer, independent of TNM stage and weight loss history. Sarcopenic obesity was an independent predictor of survival. The development of sarcopenia with aging is a multifactorial change. The body fat rate increases up to age 70 and then enters the declining phase [33]. Vertebral compression causes shortening in height which affects BMI [34]. Decreased physical activity, loss of mitochondrial volume, and fall in oxidative capacity decrease the resting metabolic rate [35, 36]. By age, DHEA sulfate, testosterone, and estrogen values reduce. The hormones maintain muscle mass [37, 38] and activation of the inflammatory pathway, playing a critical role in the development of sarcopenia [39, 40].

Zhuang et al. [41] found a significant decrease in survival in TNM stage 2 and stage 3 gastric cancer, and sarcopenia was an unfavorable prognostic factor. The increase in stage and mortality was correlated in our study. Mortality was high in both sarcopenic and non-sarcopenic patients. Albumin was associated with decreased survival in gastric cancer patients [42]. We found an association between low albumin value and mortality in patients without sarcopenia. The low albumin level of the patients who died in sarcopenic patient group was statistically nonsignificant.

The relationship between sarcopenia and obesity with cancer has recently gained importance; obesity in cancer patients is increasing parallel to the high obesity prevalence [43]. In our study, sarcopenia was lower in those with higher BMI; BMI was 0.9 times protective against mortality. The mortality rate was significantly higher in the sarcopenia + obesity group. Obesity was protective against mortality. Lee et al. [44] reported similar results to our findings; they observed a decline in disease-related survival and postoperative survival as BMI decreased. Martin et al. [45] showed that BMI predicted survival, and the heaviest patients had the longest survival time. Weight loss of more than 8% was associated with reduced survival.

Obesity is an advantage for survival since catabolism increases in cancer patients [46]. It was reported that weight loss of 15% in the first month following surgery was the only independent significant risk factor [47]. The postoperative weight loss in non-adipose body mass was critical in discontinuing adjuvant chemotherapy [48]. Early termination of treatment in patients with low BMI negatively affects the survival time. The patients who had obesity, sarcopenia, and sarcopenic obesity had gradually increased postoperative complication risks [19]. Low BMI was involved with more severe complications and poor prognosis in advanced stages. High BMI in gastric cancer patients who underwent gastrectomy showed a superior outcome compared with patients with normal BMI [49]; this concept is called the obesity paradox. The obesity paradox states that patients with obesity have advantages over normal-weight patients, contrary to the expectation that excess BMI is associated with an increased risk of death. Moreover, being overweight and having obesity does not increase the risk in some conditions; obesity may even be protective against mortality [49, 50]. Our research proved the obesity paradox; weight was a protective factor for mortality. BMI was 0.9 times protective against mortality (*p* = 0,016).

We found that sarcopenia was associated with overall survival in gastric cancer patients; sarcopenia significantly increased mortality. Since 52.5% of all patients are sarcopenic, sarcopenia should be considered a critical parameter in gastric cancer patients. The presence of obesity in the sarcopenic group was protective against mortality. Survival was less than ten months in sarcopenic patients. In a meta-analysis, sarcopenia was associated with augmented overall mortality compatible with our study [9].

Patients with sarcopenic obesity have a higher tendency to cardiovascular disease (CVD) [51]. There was a higher rate of CVD in patients with metabolic syndrome and coexistence of sarcopenia [52]. We found the lowest survival time was 27.6 ± 3.4 months in patients with DM. It was 29.3 ± 2.8 months in the presence of cardiovascular disease and 32.7 ± 3.4 months in the presence of hyperlipidemia, but it was not statistically significant.

It was shown that SMI was an independent predictor for poor prognosis in metastatic gastric cancer patients who underwent chemotherapy [53]. Mirkin et al. [54] showed that preoperative chemotherapy significantly increased the incidence of sarcopeniaand perioperative complications in patients who had chemotherapy before gastrectomy in gastric cancer. Sarcopenia and sarcopenic obesity had a relationship with the early suspension of neoadjuvant chemotherapy in resected gastric cancer [10].

Sarcopenia and sarcopenic obesity are associated with early termination of neoadjuvant chemotherapy [10]. The management of sarcopenia is critical; it increases complications and mortality. Huang et al. [55] emphasized the importance of sarcopenia classification as a determinative and independent factor among postoperative complications. They found that the worst post-gastrectomy results occurred in patients with advanced sarcopenia. Sarcopenia should be evaluated preoperatively, as it increases the complication risks [15]. Sarcopenic obesity was an independent preoperative risk factor for postoperative site infection in patients who underwent laparoscopic total gastrectomy [56]. It was shown that postoperative complications were associated with age ≥ 65 and sarcopenia but not with other parameters (BMI, cardiopulmonary comorbidity, DM, tumor characteristics, etc.) [43]. In the overweight and obesity group, the risk of postoperative complications was six times higher. We detected 64.2% of the patients had complication rates without any correlation with sarcopenia and mortality; we presumed that the tumor burden of advanced disease caused it.

Computed tomography can easily show sarcopenia and sarcopenic obesity with high accuracy for diagnosis and staging (the reported error margin is 1.4%) [57]. Our results highlighted the importance of assessing body composition integrity and interpreting it with CT imaging, which commonly provides valuable prognostic information. CT is a quick and practical method for assessing mortality in cancer patients.

Exercise and nutrition programs may stop the development of sarcopenia by increasing protein synthesis [58]. Dietary proteins prevent an inevitable decrease in muscle mass, even in old age [59]. Nutrition programs can be initiated preoperatively. Leucine amino acid, omega-3 polyunsaturated fatty acid supplementation, and exercise programs for protecting skeletal muscles and function provide synergy [43]. Protein, creatine, and β-hydroxy β-methyl butyric acid supplementation are effective for increasing muscle mass [60].

Benjamin et al. [61]found a significant decrease in the total psoas area and skeletal muscle attenuation and a lower overall survival rate in patients who underwent neoadjuvant chemotherapy compared to the operated patients. They suggested that available muscle mass is a ready-to-consume resource of the sick body and can be critical in evaluating treatment options and choosing the appropriate treatment for the body's reserve, leading to personalized therapy. Fat and muscle mass constitute a potential reserve against the stress of cancer, so it will be wise to determine the body's resources and make a survival plan accordingly. Sarcopenia and sarcopenic obesity are the risk factors for gastric cancer, and we should provide tight BMI and SMI control in those patients [62].

## Conclusions

Sarcopenia is more common in men and those ≥ 65, and is associated with higher mortality in gastric cancer. Conversely, a higher BMI has a protective role against mortality in these patients. Therefore, maintaining muscle mass and appropriate body composition is a significant step towards reducing mortality in cancer patients. Computed tomography is helpful in assessing body composition, diagnosing sarcopenia, and predicting prognosis from the onset of the disease in clinical relevance.

### Limitations

We defined sarcopenia by measuring muscle mass. Since the study was retrospective, tests measuring muscle strength, such as 'walking speed' and 'grip strength' could not be performed. A prospective study can yield more detailed results using dynamic and passive techniques.

This study has a low number of patients and an unequal distributionof gender. The patient cohort was small due to the inadequate appropriate CT images of the patients, lack of height and weight information, and follow-up of patients in other centers. A higher number of patients with an equal number of patients from both genders would have been ideal for further research.

## Data Availability

The datasets used and/or analyzed during the current study can be accessed from the corresponding author on reasonable request.

## Abbreviations

BMI: Body mass index
CVD: Cardiovascular disease
CT: Computerized tomography
SMA: Skeletal muscle area
SMI: Skeletal muscle index
